# Linking Ventilator Injury-Induced Leak across the Blood-Gas Barrier to Derangements in Murine Lung Function

**DOI:** 10.3389/fphys.2017.00466

**Published:** 2017-07-07

**Authors:** Bradford J. Smith, Elizabeth Bartolak-Suki, Bela Suki, Gregory S. Roy, Katharine L. Hamlington, Chantel M. Charlebois, Jason H. T. Bates

**Affiliations:** ^1^Department of Bioengineering, Anschutz Medical Campus, University of Colorado DenverAurora, CO, United States; ^2^Department of Biomedical Engineering, Boston UniversityBoston, MA, United States; ^3^Department of Medicine, Vermont Lung Center, Larner College of Medicine at The University of VermontBurlington, VT, United States

**Keywords:** lung injury, mechanical ventilation, ARDS, alveolar leak, surfactant dysfunction

## Abstract

Mechanical ventilation is vital to the management of acute respiratory distress syndrome, but it frequently leads to ventilator-induced lung injury (VILI). Understanding the pathophysiological processes involved in the development of VILI is an essential prerequisite for improving lung-protective ventilation strategies. The goal of this study was to relate the amount and nature of material accumulated in the airspaces to biomarkers of injury and the derecruitment behavior of the lung in VILI. Forty-nine BALB/c mice were mechanically ventilated with combinations of tidal volume and end-expiratory pressures to produce varying degrees of overdistension and atelectasis while lung function was periodically assessed. Total protein, serum protein, and E-Cadherin levels were measured in bronchoalveolar lavage fluid (BALF). Tissue injury was assessed by histological scoring. We found that both high tidal volume and zero positive end-expiratory pressure were necessary to produce significant VILI. Increased BALF protein content was correlated with increased lung derecruitability, elevated peak pressures, and histological evidence of tissue injury. Blood derived molecules were present in the BALF in proportion to histological injury scores and epithelial injury, reflected by E-Cadherin levels in BALF. We conclude that repetitive recruitment is an important factor in the pathogenesis of VILI that exacerbates injury associated with tidal overdistension. Furthermore, the dynamic mechanical behavior of the injured lung provides a means to assess both the degree of tissue injury and the nature and amount of blood-derived fluid and proteins that accumulate in the airspaces.

## Introduction

Mechanical ventilation is necessary for managing the respiratory failure that defines acute respiratory distress syndrome (ARDS) (Force et al., 2012). However, mechanical ventilation itself can cause further damage to an already injured alveolar blood-gas barrier (Thammanomai et al., 2013). This allows additional leak of blood-derived materials into the airspaces where they impair surfactant function and raise surface tension (Holm and Notter, 1987; Holm et al., 1988; Günther et al., 1996), thereby exacerbating the injurious stresses caused by the ventilation. Avoiding ventilator-induced lung injury (VILI) thus requires ventilating in a way that avoids damaging the alveolar barrier (Amato et al., 1998). Achieving this in practice in any given ARDS lung, however, is challenging because it is not possible to determine alveolar leak directly with the rapidity required to make appropriate adjustments to ventilator strategy before injury worsens. On the other hand, measurements of lung mechanics can be made in an ongoing fashion with the necessary rapidity (Terragni et al., 2003), and thus have the potential to guide the application of mechanical ventilation in ARDS provided they can be linked to events occurring at the level of the alveolar leak.

We have previously shown in mice that accelerating VILI can be induced by several hours of mechanical ventilation using a high tidal volume (Vt) and a positive end-expiratory pressure (PEEP) of zero (Seah et al., 2011). We have also shown that the rate of VILI development achieved can be titrated through adjustment of Vt, making this a convenient model for studying precisely how VILI depends on the parameters of mechanical ventilation (Smith et al., 2013). However, while VILI produced in this way seems likely to involve accumulation of blood-derived material in the lung airspaces (Nin et al., 2008; Seah et al., 2011), we have yet to ascertain the temporal correlation between such accumulation and the developing derangements in lung function. We also do not know how the makeup of the leaked material affects lung function. Gaining this information would greatly strengthen our ability to use measurements of lung function as surrogate measures of the pathophysiological processes underlying VILI.

Accordingly, the aim of the present study was to determine the link between key parameters of injurious mechanical ventilation, the derangements in lung function that result, and the nature of the leaked material that accumulates in the lung airspaces over time. We hypothesize that alterations in lung function reflect the amount of proteinaceous material that has accumulated in the airspace, and thus provide immediate feedback on the integrity of the alveolar wall. By establishing this hypothesis in the present study, we will set the scene for the use of lung function measurements as a means of rapidly and continually tracking the progress of VILI so that minimally injurious ventilation strategies can be identified.

## Methods

### Animal preparation

This study was carried out in accordance with the recommendations of the Institutional Animal Care and Use Committee of the University of Vermont and was in compliance with the Animal Welfare Act. The protocol was approved by the Institutional Animal Care and Use Committee of the University of Vermont. Healthy 10–12 weeks old BALB/c mice (Jackson Laboratories, Bar Harbor, ME) weighing 18.1–24 g (average 20.1 g) were anesthetized with 90 μg/kg intraperitoneal (IP) sodium pentobarbital, tracheostomized, and ventilated using a flexiVent small animal ventilator (SCIREQ, Montreal, QC, Canada). To prevent spontaneous breathing efforts from corrupting measurements of lung function, 0.8 mg/kg IP pancuronium bromide was administered at the onset of ventilation; heart rate was continuously monitored via EKG to ensure appropriate anesthesia depth. Five microgram per kilogram of IP sodium pentobarbital and 0.15 ml IP 5% dextrose lactated ringers solution were administered at 30 min intervals. No spontaneous breathing efforts were detected throughout the experiment, and no animals exhibited increased heart rate indicative of inadequate anesthesia.

### Study protocol

After the preparation protocol, the derecruitability of the lungs in each mouse was determined as follows. First, a deep inspiration (DI) consisting of a 3 s ramp to 30 cmH_2_O followed by a 3 s breath hold was applied to recruit the lungs. Three min of mechanical ventilation was then applied with tidal volume Vt = 10 ml/kg, frequency *f* = 200 breaths/min, and positive end-expiratory pressure PEEP = 0 cmH_2_O. Every 20 s during this period of ventilation the impedance of the lung was measured at frequencies from 0.5 to 20.5 Hz using a broad-band perturbation from the flexiVent piston (peak-peak amplitude 0.17 ml). Each impedance was fit with the constant-phase model (Hantos et al., 1992) in order to determine lung elastance (*H*) (Schuessler and Bates, 1995; Allen et al., 2002, 2007; Allen and Bates, 2004; Seah et al., 2011). A total of 9 measurements of *H* were made in this way. This procedure (DI followed by 9 impedance measurements) was then repeated for PEEP = 3 and 6 cmH_2_O.

As listed in Table 1, the animals in five treatment groups received various combinations of Vt and PEEP for 100 min of mechanical ventilation that was interrupted at regular 5 min intervals by a 20 s measurement period. This period began with 18 s of ventilation with a tidal volume Vt_PV_ (Table 1) to standardize the volume history of the lungs and was followed by the measurement of a 1 Hz pressure-volume (PV) loop using a volume amplitude of Vt_PV_. The value of Vt_PV_ was reduced in the PEEP = 3 cmH_2_O groups (Vt_PV_ = 42.5 ml/kg) compared to the PEEP = 0 groups (Vt_PV_ = 45 ml/kg) so that lung volumes at end-inspiration were comparable. At the conclusion of the 100 min period of ventilation the mice were briefly removed from the ventilator and received a retro-orbital injection of 25 mg/kg fluorescein isothiocyanate-labeled 4 kDa dextran (FITC-D, Sigma-Aldrich, St. Louis, MO) diluted to 5 mg/ml in phosphate-buffered saline (PBS). After being reattached to the ventilator the mice received two DIs separated by 30 s of ventilation (Vt = 10 ml/kg and PEEP = 3 cmH_2_O) and a second round of derecruitability tests (described above) were performed. Control animals (*n* = 7) received a FITC-D injection and then one round of derecruitability tests before harvest.

**Table 1:** Ventilation parameters during 100 min mechanical ventilation period.

**Group**	***n***	**Vt(ml/kg)**	**PEEP (cmH_2_O)**	***f* (breaths/min)**	**Vt_PV_ (ml/kg)**
Control	7	No ventilation, derecruitment test only			
Low-Vt/PEEP3	10	10	3	200	42.5
Low-Vt/PEEP0	9	10	0	200	45
Mid-Vt/PEEP3	9	42.5	3	50	42.5
Mid-Vt/PEEP0	9	45	0	50	45
High-Vt/PEEP0	12	55	0	50	45

*Vt, tidal volume; PEEP, positive end-expiratory pressure; f, ventilation frequency; Vt_pv_, tidal volume during pressure-volume loop measurement. Note that fewer animals were included in the Control group because the pulmonary system elastance variability in this group was less than in the study groups. The differences in the other group numbers (n) were due to variable but low drop-out of animals not surviving the experiment*.

### Sample collection

Following mechanical ventilation the animals were removed from the ventilator and bronchoalveolar lavage fluid (BALF) was collected by instilling 1 ml of PBS through the tracheal cannula and suctioning back ~0.9 ml. The fluid was centrifuged for 10 min at 1,600 rpm, and the supernatant was preserved for analysis. Blood was collected via cardiac puncture and centrifuged for 10 min at 2,400 rpm. BALF supernatant total protein content was measured using a BCA protein assay kit (Pierce, Rockford, IL). The relative concentrations of FITC-D in the BALF and serum were determined using a fluorescent plate reader (Bio-Tek Synergy HTX, Winooski, VT, USA) as we have previously described (Allen et al., 2007).

### Histology

Excised lungs were fixed via intratracheal instillation of 10% neutral buffered formalin at a pressure of 20 cmH_2_O. The lungs were embedded in paraffin and 5 lungs from each experimental group were randomly selected for histological scoring. Coronal sections (7 μm thick) were obtained and deparaffinized in xylene and rehydrated using a series of water/alcohol solutions of progressively decreasing alcohol fraction. Hematoxylin and eosin (H&E) staining was performed using the Protocol Reagents (Fisher Scientific). Histological analysis was carried out blindly using light microscopy (Nikon Eclipse 50i microscope and SPOT camera, Micro Video Instruments) at low (10X) and high (40X) magnifications for 20 randomly selected sections from each mouse. The degree of VILI in whole sections was scored using criteria derived from previous histological investigations by us and others (Muscedere et al., 1994; Nin et al., 2008; Hamakawa et al., 2011; Szabari et al., 2012). Specifically, scores of 0–10 were assigned for each of the following characteristics:

1. *Alveolar wall thickening* accompanied by structural changes of the alveolar extracellular matrix (ECM) due to increased volume and/or loose ECM and of the alveolar layer due to hyaline membrane formation covering the epithelium or vacuolar necrotic epithelial cell damage;
2. *Alveolar wall rupture* with thinner or broken alveolar walls and tissue retraction;
3. *Blood cell infiltration* including macrophages, monocytes, lymphocytes, and neutrophils with the increase of inflammatory cell numbers in the alveolar wall space;
4. *Interstitial edema* with proteinaceous fluid and swelling of the ECM of the alveolar septa;
5. *Vascular bed injury* including capillary dilation and congestion with the degeneration events of vessels (swollen, vacuole rich vascular walls with cell necrosis); and
6. *Alveolar wall flooding* reflecting platelet invasion including infarcts like hemorrhagic changes of alveolar walls after vascular breakage.

### Sample processing for western blot and gel staining

Relative changes of immunoglobulin G heavy (IgG-HC) and light (IgG-LC) chains, serum albumin, and soluble E-cadherin were determined in the BALF of 6 randomly selected mice from each group. Protein concentration was determined using the BCA protein assay kit (Pierce, Rockford, IL). Equal amounts (3.7 μg) of samples were normalized to equal volumes and were separated using 4–20% SDS-polyacrylamide gels (Bio-Rad Laboratories), transferred onto polyvinylidene fluoride membranes (Millipore, Bedford, MA), and blocked overnight with 5% bovine serum albumin in phosphate buffered saline/0.05% Tween 20. Western blot analysis was carried out with mouse, goat, or rabbit primary antibodies for mouse serum albumin (1 μg/ml), IgG-LC (1:2,000), IgG-HC (1:8,000), and E-cadherin (1:1,000) to assess the levels of these proteins in the BALF. All primary and secondary antibody incubation was done for 1 h and all antibodies were from Abcam Inc. (Cambridge, MA). Quantitative densitometry was performed after chemiluminescence detection using SuperSignalWest Pico chemiluminescence substrates (Pierce). Coomassie G-250 blue staining (Bio-Rad, Hercules, CA) was used to visualize the protein species in gels after electrophoresis as above. Briefly, gels were washed in water 3 times for 5 min and stained for 1 h followed by a 30 min water wash. Quantitative densitometry was performed for the large 150–250 kDa, medium 65–70 kDa, and small 10–15 kDa proteins.

### Pressure-volume loop analysis

**Figure 1:**
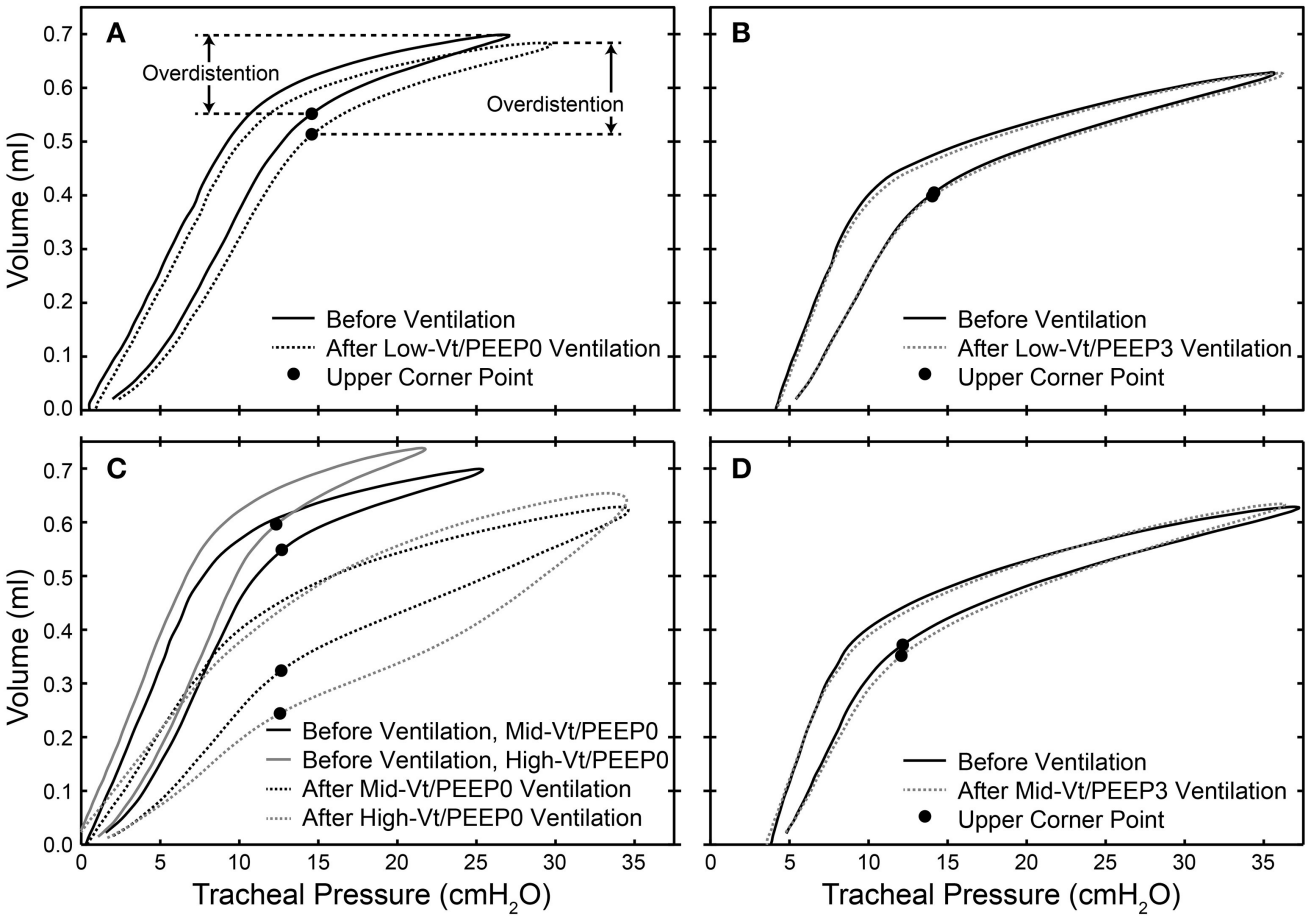
Dynamic pressure-volume loops showing the location of the upper corner point determined by fitting to the Venegas equation (filled circles) and the overdistension volume before (solid lines) and after (dotted lines) 100 min of ventilation for representative mice in the Low-Vt/PEEP0 **(A)**, Low-Vt/PEEP3 **(B)**, Mid-Vt/PEEP0 **(C)**, High-Vt/PEEP0 **(C)**, and Mid-Vt/PEEP3 **(D)** groups.

Dynamic pressure-volume loops recorded during the 100 min period of ventilation were analyzed using MATLAB (Mathworks, Natick, MA, USA) in order to determine the peak level of distension applied during ventilation. For each animal we determined the pressure at which the upper corner point is located (*P*_*Corner*_) by fitting the Venegas equation (Venegas et al., 1998). The location of the corner points for representative mice are shown with filled circles in Figure 1. The ratio of the ventilator cylinder displacement to the delivered volume at peak inspiration was calculated and used to compute the volume delivered for each breath during the 5 min epochs that comprised the 100 min of ventilation because the flexiVents used in this experiment deliver ventilation based on a prescribed cylinder displacement. The volume delivered above the upper corner point was multiplied by the duration of the ventilation epoch and the ventilation frequency to estimate the total volume delivered above the upper corner point for each epoch. These volumes were summed over the course of the experiment in order to provide the total volume delivered above the upper corner point, which we take as a measure of the total degree of overdistension (Σε_T_).

### Statistical analysis

Changes in peak pressure during the dynamic pressure-volume loops recorded during ventilation were fit to a linear model and the difference in slope between groups was compared using Welch one-way ANOVA and Games–Howell *post-hoc* test. The equation *H* = *H*_1_ *t*^β^ was fit to the nine elastance measurements recorded during derecruitability tests at PEEP = 0, 3, and 6 cmH_2_O. *H*_1_ represents the elastance magnitude, β indicates the rate of *H* increase, and *t* is the time in the derecruitability test in seconds. Post-ventilation differences in *H*_1_ and β between ventilation treatment groups at each derecruitment test PEEP were tested using Welch one-way ANOVA and Games–Howell *post-hoc* test (assumption of homogeneity of variances was violated in each case). Differences in *H*_1_ and β during derecruitment tests at PEEP = 0, 3, and 6 cmH_2_O within each ventilation group were tested using one-way repeated measures ANOVA and *post-hoc* multiple comparisons with Bonferroni adjustment. If the assumption of sphericity was violated, the Greenhouse-Geisser correction was used. The differences in *H*_1_ and β before and after ventilation within each ventilation treatment group at each derecruitability test PEEP were assessed with paired *t-*tests. One-way ANOVA was used for assessment of western blots for specific proteins and for gel staining. When the normality test failed, corresponding non-parametric statistical procedures were used. *Post-hoc* comparisons included Holm–Sidak and Tukey tests for one-way parametric ANOVA and non-parametric ANOVA (Kruskal–Wallis), respectively. Statistical significance was accepted at *p* < 0.05.

## Results

In the High-Vt/PEEP0 group the ventilator cylinder displacement was set to Vt = 55 ml/kg with the average delivered Vt = 44 ml/kg due to gas compression in the ventilator circuit. The Mid-Vt/PEEP0 animals received an average delivered Vt = 35 ml/kg, the Mid-Vt/PEEP3 average delivered Vt = 32 ml/kg, and the Low-Vt average delivered Vt = 10 ml/kg. These resulted in minute ventilations for the various groups of: High-Vt/PEEP0 = 2,200 ml/kg/min; Mid-Vt/PEEP0 = 1,750 ml/kg/min; Mid-Vt/PEEP3 = 1,600 ml/kg/min; Low-Vt = 2,000 ml/kg/min.

**Figure 2:**
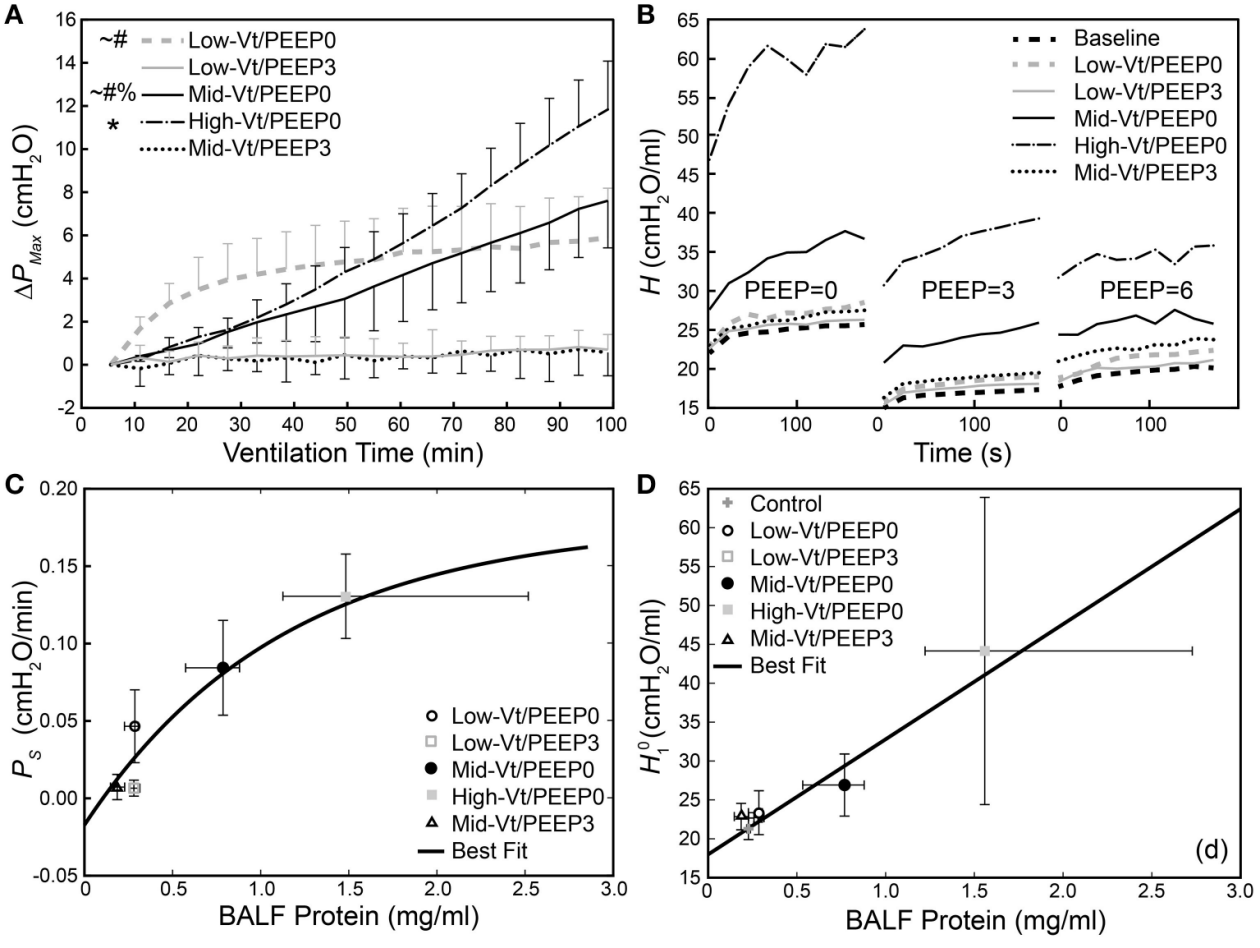
**(A)** Change in dynamic pressure-volume loop peak pressure (**Δ***P*_*Max*_) during ventilation. ^*^Indicates that the rate of increase of Δ*P*_*Max*_ (*P*_*S*_) was significantly greater than all other groups; significant increases from Mid-Vt/PEEP3 (#), Low-Vt/PEEP3 (~), and Low-Vt/PEEP0 (%). Error bars show standard deviation. **(B)** Changes in derecruitment dynamics at PEEP = 0, 3, and 6 cm H_2_O caused by mechanical ventilation. The statistical significance of intra-group elastance changes over the course of ventilation are shown in Table 2, intra-group differences in elastance at different derecruitment test PEEPs are shown in Table 3, and post-ventilation inter-group elastance changes are shown in Supplementary Tables 2–4. **(C)** *P*_*S*_ increases as a function of BALF total protein [*y* = 0.18(1 – *e*^−0.89(x-0.10)^), *R*^2^ = 0.81, *p* < 0.027, black line]. **(D)** Increase in initial elastance during the PEEP = 0 derecruitability test ($H_{1}^{0}$**)** at the conclusion of the experiment is correlated with increased BALF total protein (*y* = 18.0 + 14.1x, *R*^2^ = 0.78, *p* < 10^−18^). Data points for $H_{1}^{0}$ and *P*_*S*_ show the mean with error bars showing standard deviations; data points for BALF protein show the median with error bars showing the interquartile range.

Figure 2A shows that the change in peak airway pressure (Δ*P*_*Max*_) evolved in different ways throughout the 100 min of mechanical ventilation in the five treatment groups. Peak airway pressures (*P*_*Max*_) are shown in Supplementary Figure 1. Minimal changes in Δ*P*_*Max*_ were observed in the two groups ventilated with PEEP = 3 cmH_2_O (Low-Vt/PEEP3 and Mid-Vt/PEEP3), whereas substantial and progressive increases were observed in the two groups receiving moderate and high Vt at zero PEEP (Mid-Vt/PEEP0 and High-Vt/PEEP0). The remaining group ventilated with a low Vt at PEEP = 0 (Low-Vt/PEEP0) exhibited an initial rapid increase in Δ*P*_*Max*_ that transitioned to a much slower rate of increase at about 30 min. Figure 2B shows, however, that the post-ventilation derecruitability test measurements of respiratory elastance *H* were clustered somewhat differently among the five treatment groups. The substantial increases in Low-Vt/PEEP0 Δ*P*_*Max*_ were not reflected in the post-ventilation derecruitability tests that were preceded by a recruitment maneuver.

The equation *H* = *H*_1_ *t*^β^ was fit to elastance measurements recorded over times *t* in the derecruitability tests. Superscripts on the initial elastance (*H*_1_) and elastance increase rate (β) demarcate the derecruitment test PEEP (e.g., $H_{1}^{3}$ and β^3^ correspond to a derecruitment test performed at PEEP = 3 cmH_2_O). The mean and standard deviation of *H* as well as the average fit for each group are shown in Supplementary Figure 2. Significant increases in *H*_1_ between the pre- and post-ventilation derecruitability tests at PEEP = 0, 3, and 6 cmH_2_O were observed in the Mid- and High-Vt/PEEP0 groups (Table 2). A small but statistically significant increase in $H_{1}^{3}$ was also present in the Low-Vt/PEEP0 group. Increases in β^0^ and β^3^ were observed in both Mid- and High-Vt/PEEP0. β^6^ increased in the Low-Vt/PEEP0 group, β^3^ increased in the Mid-Vt/PEEP3 group, and β^0^ increased in the Low-Vt/PEEP3 group. The mean and 95% confidence intervals (CI) of the changes in derecruitability test elastance are provided in Table 2.

**Table 2:** Comparison of pre- and post-ventilation elastance measured during PEEP = 0, 3, and 6 cmH_2_O derecruitability tests for each ventilation group showing mean differences [95% CI] in initial elastance (*H*_1_) and in the elastance increase rate (β).

		**PEEP = 0**	**PEEP = 3**	**PEEP = 6**
High-Vt/PEEP0	*H_1_*	22.34 [9.84, 34.84]^*^	15.08 [8.93, 21.23]^*^	15.12 [9.77, 20.47]^*^
	β	0.047 [0.025, 0.069]^*^	0.019 [0.010, 0.028]^*^	0.002 [−0.007, 0.011]
Mid-Vt/PEEP0	*H_1_*	4.98 [2.23, 7.72]^*^	4.90 [3.71, 6.10]^*^	5.35 [2.18, 8.83]^*^
	β	0.031 [0.013, 0.048]^*^	0.020 [0.003, 0.036]^*^	0.001 [−0.008, 0.011]
Low-Vt/PEEP0	*H_1_*	2.17 [0.49, 3.85]^*^	1.22 [−0.41, 2.84]	1.38 [−0.89, 3.65]
	β	0.004 [-0.004, 0.012]	0.009 [0.000,0.017]	0.007 [0.004, 0.010]^*^
Mid-Vt/PEEP3	*H_1_*	−0.046 [−1.42, 0.50]	0.52 [−0.38, 1.43]	−0.01 [−2.25, 2.22]
	β	0.004 [−0.004, 0.011]	0.006 [0.001, 0.011]^*^	−0.006 [−0.020, 0.007]
Low-Vt/PEEP3	*H_1_*	−0.15 [−0.69, 0.40]	0.19 [−0.16, 0.53]	0.63 [−0.42, 1.69]
	β	0.004 [0.001, 0.007]^*^	0.001 [−0.003, 0.005]	−0.004 [−0.010, 0.003]

* *Shading indicates statistical significance at p < 0.05*.

The post-ventilation differences in *H*_1_ and β between treatment groups during PEEP = 0, 3, and 6 cmH_2_O derecruitability tests are shown in Supplementary Tables 2–4, respectively. Post-ventilation values of *H*_1_ and β are shown in Supplementary Tables 5,6. *H*_1_ in the High-Vt/PEEP0 group was significantly greater than the Low-Vt/PEEP0, Mid-Vt/PEEP3, and Low-Vt/PEEP3 groups at all derecruitment test PEEPs. $H_{1}^{3}$ was greater for the High-Vt/PEEP0 group than the Mid-Vt/PEEP0 animals, and $H_{1}^{6}$ increased in the Mid-Vt/PEEP0 group with respect to Low-Vt/PEEP3. Intragroup post-ventilation changes in β were more pronounced in the PEEP = 0 derecruitability tests with the High-Vt/PEEP0 group demonstrating greater β^0^ than Low-Vt/PEEP0, Mid-Vt/PEEP3, and Low-Vt/PEEP3. The High-Vt/PEEP0 β^3^ increased in comparison to Low-Vt/PEEP3.

Differences within each treatment group in *H*_1_ and β between derecruitability tests conducted at PEEP = 0, 3, and 6 cmH_2_O are detailed in Table 3. Prior to ventilation $H_{1}^{6}$ was significantly greater than $H_{1}^{3}$; $H_{1}^{0}$ was significantly greater than both $H_{1}^{3}$ and $H_{1}^{6}$. There were no significant differences in β between PEEPs prior to ventilation. These alterations in *H*_1_ were maintained post-ventilation in the Low-Vt/PEEP3 group and were accompanied by a small but significant increase in β^3^ with respect to β^6^. Mid-Vt/PEEP3 ventilation was characterized by differences between $H_{1}^{0}$ and $H_{1}^{3}$ as well as $H_{1}^{3}$ and $H_{1}^{6}$. Low-Vt/PEEP0 ventilation resulted in significant differences between $H_{1}^{0}$ and $H_{1}^{3}$ in addition to $H_{1}^{0}$ and $H_{1}^{6}$. In the Mid- and High-Vt/PEEP0 groups β was different at all derecruitment test PEEPs and $H_{1}^{0}$ was greater than $H_{1}^{3}$. The High-Vt/PEEP0 animals also demonstrated greater $H_{1}^{0}$ than$H_{1}^{6}$.

**Table 3:** Effect of PEEP during derecruitment tests within ventilation treatment groups.

		**PEEP = 0 – PEEP = 3**	**PEEP = 0 – PEEP = 6**	**PEEP = 3 – PEEP = 6**
High-Vt/PEEP0	*H_1_*	14.27 [5.07, 23.47]^*^	12.84 [1.92, 23.76]^*^	−1.43 [−5.26, 2.40]
	β	0.031 [0.009, 0.053]^*^	0.046 [0.019, 0.073]^*^	0.015 [0.003, 0.026]^*^
Mid-Vt/PEEP0	*H_1_*	7.46 [4.04, 10.87]^*^	2.66 [−6.34, 11.66]	−4.80 [−11.31, 1.72]
	β	0.016 [0.003, 0.028]^*^	0.037 [0.016, 0.057]^*^	0.021 [0.004, 0.039]^*^
Low-Vt/PEEP0	*H_1_*	7.61 [6.38, 8.83]^*^	4.68 [0.84, 8.52]^*^	−2.93 [−5.87, 0.02]
	β	0.001 [−0.006, 0.007]	0.003 [−0.009, 0.015]	0.002 [−0.009, 0.014]
Mid-Vt/PEEP3	*H_1_*	6.53 [5, 8.06]^*^	2.17 [−1.00, 5.34]	−4.36 [−7.18, −1.54]^*^
	β	0.001 [−0.003, 0.004]	0.011 [−0.002, 0.025]	0.011 [−0.003, 0.025]
Low-Vt/PEEP3	*H_1_*	7.29 [6.24, 8.34]^*^	4.47 [2.48, 6.46]^*^	−2.82 [−4.37, −1.27]^*^
	β	−0.002 [−0.007, 0.003]	0.003 [−0.003, 0.010]	0.006 [0.000, 0.011]^*^
All Groups Pre-Vent	*H_1_*	7.08 [6.58, 7.58]^*^	4.52 [3.53, 5.50]^*^	−2.57 [−3.63, −1.50]^*^
	β	0.002 [0.000, 0.005]	0.002 [−0.002, 0.006]	0.000 [−0.003, 0.003]

* *Shading indicates statistical significance at p < 0.05. Mean differences [95% CI] in initial elastance (H_1_) and in the elastance increase rate (β) between derecruitability tests conducted at PEEP = 0, 3, and 6 cmH2O before ventilation (All Groups) or at the conclusion of ventilation for each group*.

The rate of increase in Δ*P*_*Max*_ (*P*_*S*_) was determined by fitting a first-order polynomial to the pressure-volume loop peak pressures shown in Figure 2A. A monotonic relationship, shown in Figure 2C, exists between BALF protein and the degradation of lung function described by *P*_*S*_. Figure 2D presents a similar picture with respect to $H_{1}^{0}$, the initial value of *H* seen in Figure 2B. Specifically, the High-Vt/PEEP0 group produced the greatest levels of BALF protein and the highest values of both *P*_*S*_ and $H_{1}^{0}$. The Mid-Vt/PEEP0 group had the next highest values, and the apparently non-injured groups Low-Vt/PEEP3 and Mid-Vt/PEEP3 had the lowest levels of BALF protein, *P*_*S*_, and $H_{1}^{0}$. An exception to this general picture is the Low-Vt/PEEP0 group, which had elevated values of *P*_*S*_ but low levels of both $H_{1}^{0}$ and BALF protein.

**Figure 3:**
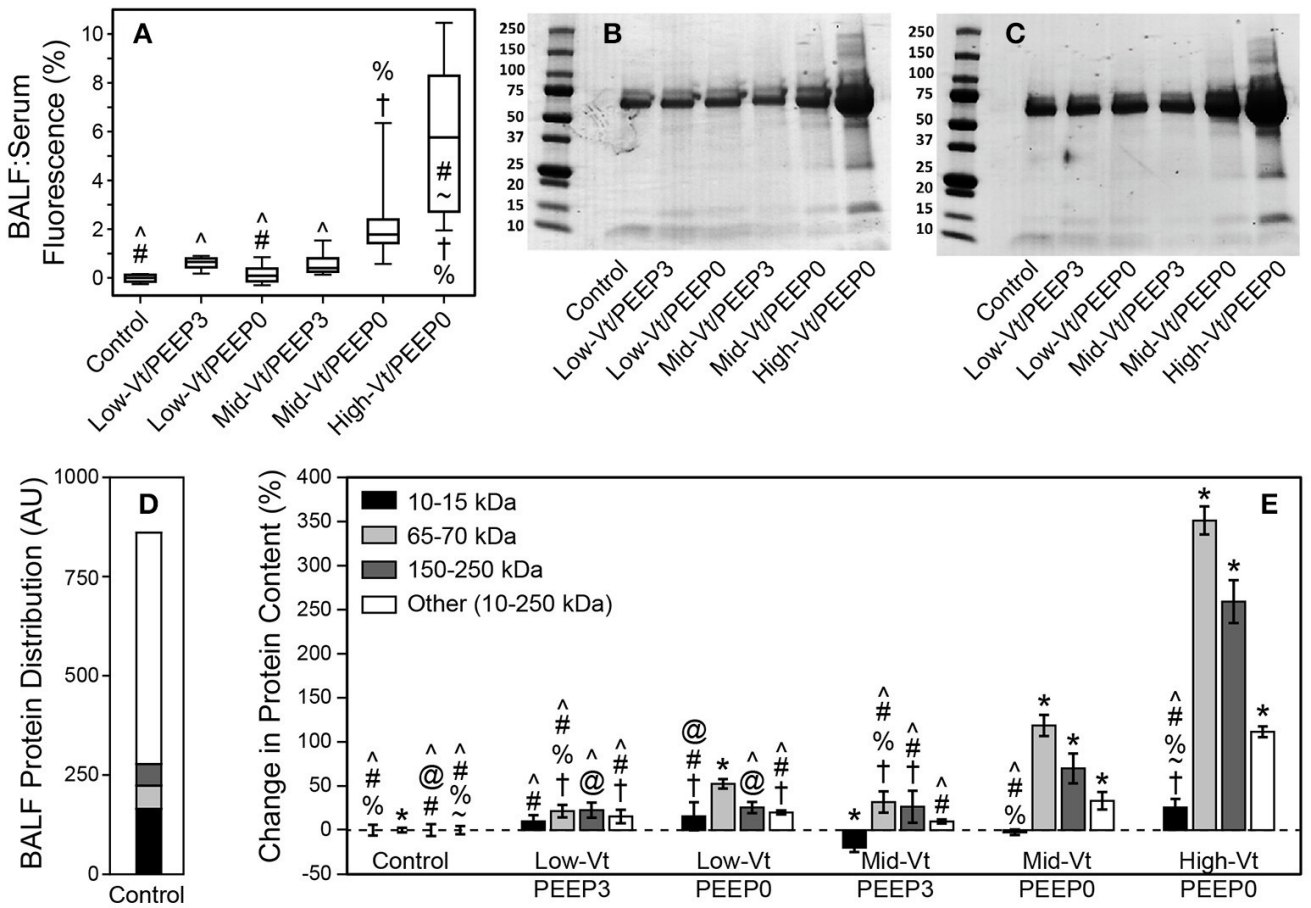
**(A)** Ratio of bronchoalveolar lavage fluid (BALF):Serum fluorescense demonstrates significantly increased leak (with respect to the control group) of retro-orbitally injected 4 kDa dextran molecules into airspace. The fluorescence ratio was linearly related to BALF protein (*y* = 0.112 + 0.269x, *R*^2^ = 0.92, *p* < 0.005). Panels **(B,C)** show representitave Coomassie G-250 blue staining used for quantitative densitometry. **(D)** Distribution of small (10–15 kDa, black), medium (65–70 kDa, light gray), large (150–200 kDa, dark gray), and all other protein species in the 10–250 kDa range (white) in the BALF as determined by densitometric analysis for the control group. **(E)** Percent changes in the protein content for each ventilation group compared to control showing significant increases in medium and large proteins caused by Mid-Vt/PEEP0 and High-Vt/PEEP0 ventilation. Significant difference from all other groups (^*^), from control (†), Low-Vt/PEEP3 (~), Low-Vt/PEEP0 (%), Mid-Vt/PEEP3 (#), Mid-Vt/PEEP0 (@), and High-Vt/PEEP0 (^∧^). Error bars show the standard deviation.

The permeability of the blood-gas barrier to very small molecules (4 kDa FITC-labeled dextran) increased markedly with more injurious ventilation, as shown by the ratio of BALF fluorescence relative to that of serum (Figure 3A), as did the amount of BALF proteins of larger sizes (Figures 3B,C). Figure 3D shows the relative amounts of proteins of different sizes in the BALF from the Control group. Figure 3E shows that the amounts of these various components were all increased by injurious mechanical ventilation in a dose-dependent fashion, with the exception of the smallest proteins (10–15 kDa), which remained essentially unchanged.

**Figure 4:**
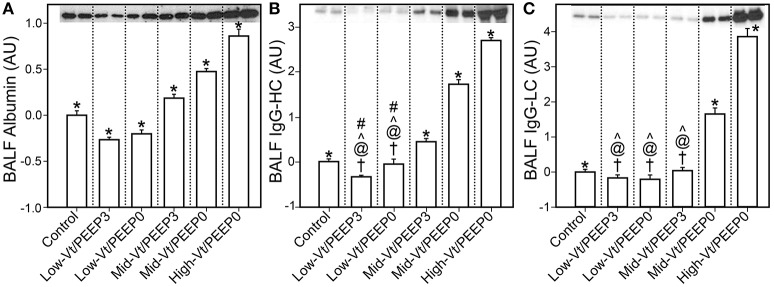
Blood-borne molecules in the bronchoalveolar lavage fluid (BALF) normalized to the control group: **(A)** serum albumin, **(B)** immunoglobulin G heavy chain (IgG-HC), **(C)** and immunoglobulin G light chain (IgG-LC). Error bars show the standard deviation. Significant difference from all other groups (^*^), from control (†), Mid-Vt/PEEP3 (#), Mid-Vt/PEEP0 (@), and High-Vt/PEEP0 (^∧^). Error bars show standard deviation.

**Figure 5:**
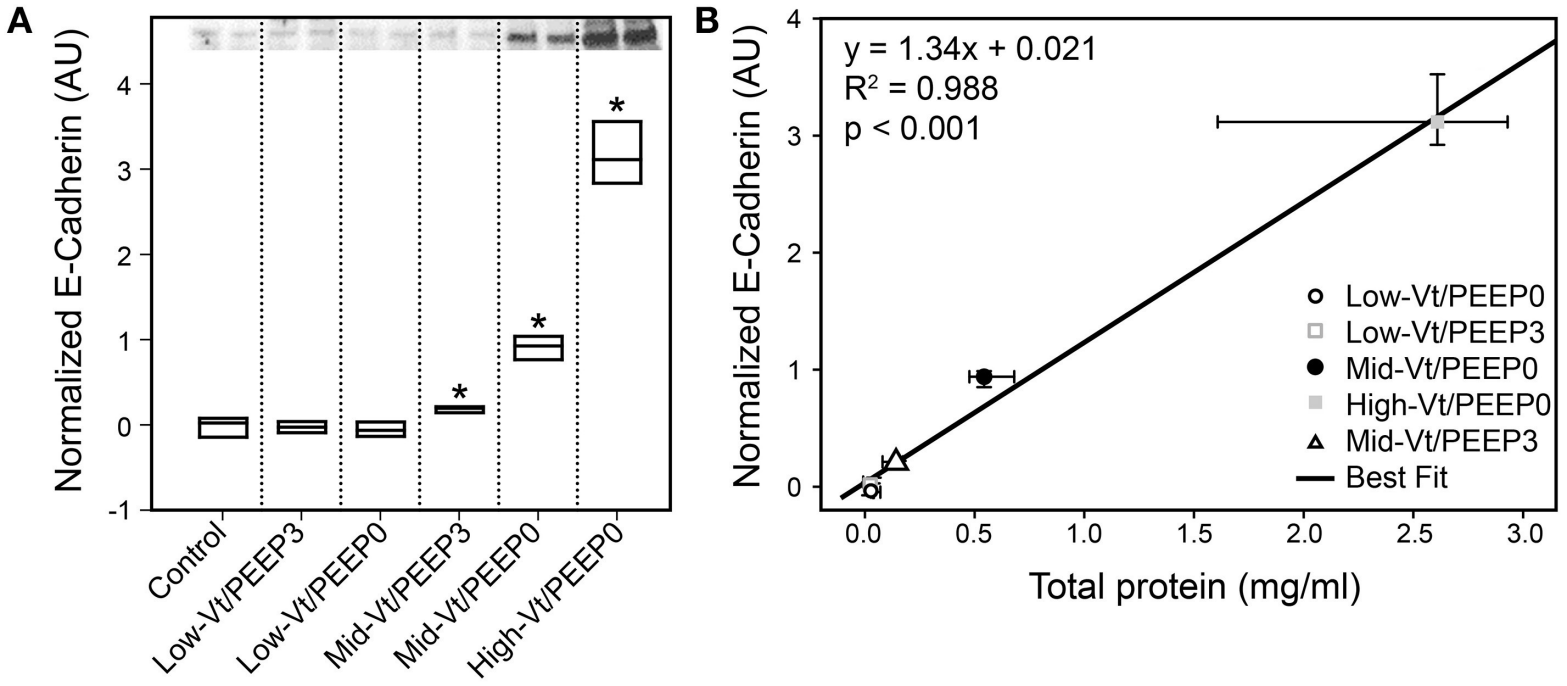
Epithelial damage marker soluble E-Cadherin content in bronchoalveolar lavage fluid (BALF). **(A)** The 85 kDa E-Cadherin level is normalized to the control group; Mid-Vt/PEEP0, Mid-Vt/PEEP3, and High-Vt/PEEP0 are significantly different from all other groups (^*^). **(B)** BALF E-Cadherin is linearly related to BALF total protein (*R*^2^ = 0.996). Data points show the median with error bars indicating the interquartile range.

Figure 4 shows that specific plasma proteins passed into the BALF in a manner that depended on the injuriousness of the ventilation regimen. Specifically, ventilation with both large tidal volumes and zero PEEP produced the greatest increases in albumin (≈66 kDa, Figure 4A), IgG-HC (≈50 kDa, Figure 4B), and IgG-LC (≈25 kDa, Figure 4C). Substantial increases in soluble E-Cadherin, a marker of epithelial damage, were observed in the BALF of both the Mid-Vt/PEEP0 and High-Vt/PEEP0 groups, with a four-fold increase in the High-Vt/PEEP0 group compared to the Control group (Figure 5A). The Mid-Vt/PEEP3 group also showed a small but significant increase. The levels of E-Cadherin in the BALF were linearly related to BALF total protein (Figure 5B), indicating that the degree of damage to the epithelial barrier is correlated with the accumulation of protein in the airspace.

**Figure 6:**
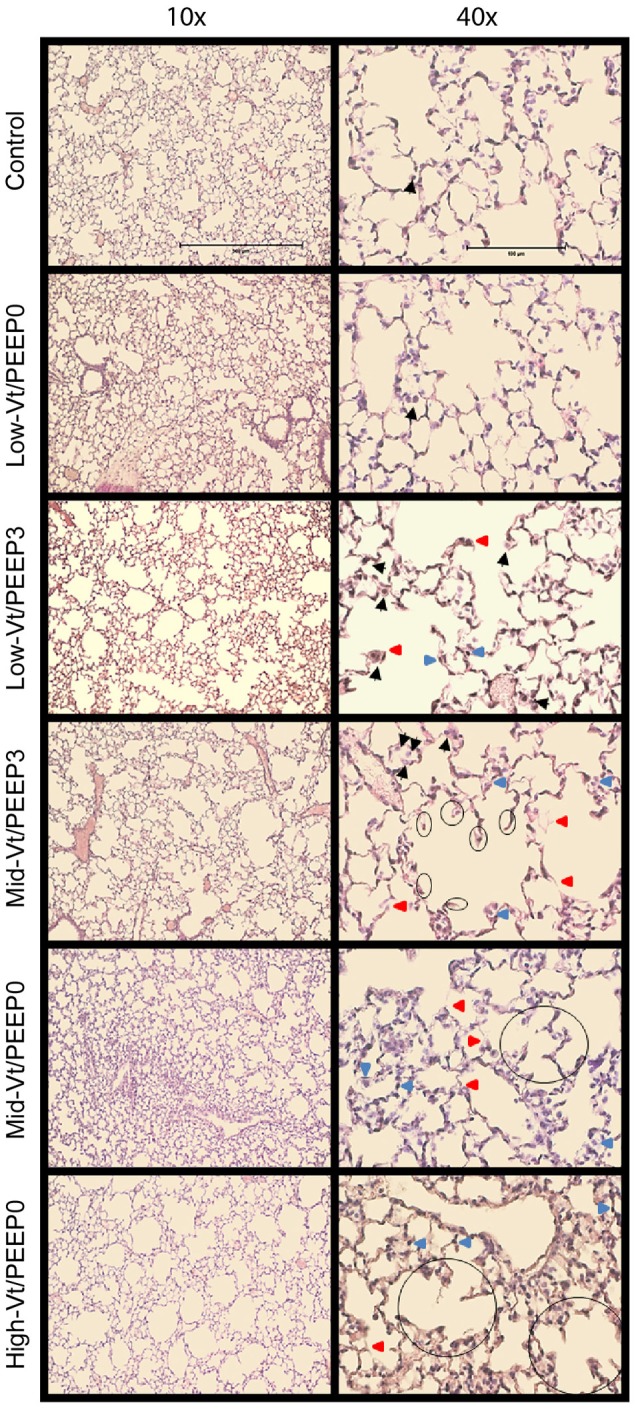
Representative microphotographs of H&E stained mouse lung sections at 10x and 40x magnifications. Black arrows point to macrophages, red arrows point to weakened, damaged, and thinner alveolar walls, and blue arrows point to dilated or congested capillaries. The already broken walls with retracted tissue are circled.

**Figure 7:**
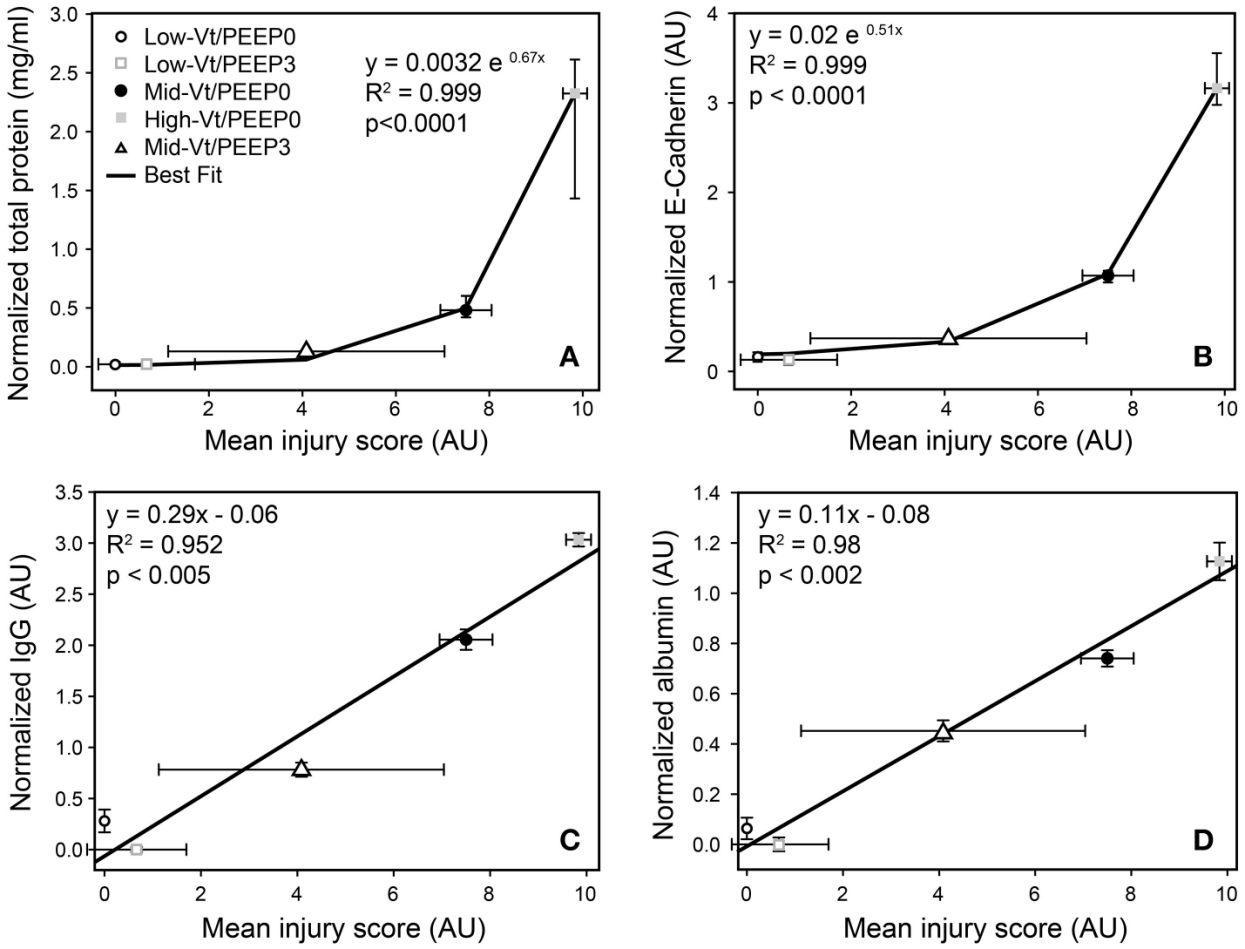
Correlation between injury scores obtained from histological evaluation and total protein **(A)**, normalized E-Cadherin **(B)**, normalized IgG-HC **(C)**, and normalized serum albumin **(D)**. Normalized total protein and E-Cadherin show the median and interquartile range; all other data are mean and standard deviation.

Blinded histological scoring revealed that low-Vt ventilation resulted in markedly less tissue damage compared to all other ventilation regimens (Table 4 and Figure 6). Even in the Mid-Vt/PEEP3 group we observed instances of alveolar wall rupture, interstitial edema, and swelling of the extracellular matrix. This structural damage increased with the removal of PEEP (Mid-Vt/PEEP0 group) and was exacerbated by increasing tidal volume (High-Vt/PEEP0 group). Supplementary Figures 3, 4 show representative images from these two groups. Neutrophils, lymphocytes, and monocytes were also observed in the parenchyma and were found with increasing frequency in the Mid-Vt/PEEP3, Mid-Vt/PEEP0, and High-Vt/PEEP0 groups, again supporting the conclusion that these groups received increasingly injurious ventilation. The two most injured groups (Mid-Vt/PEEP0 and High-Vt/PEEP0) demonstrated increasing markers of vascular bed injury that included swollen alveolar walls due to interstitial edema, capillary congestion and dilation, and alveolar flooding with blood cells. The mean of these separate injury scores was determined as an overall measure of tissue damage and was linearly related to the levels of IgG-HC (*R*^2^ = 0.952, Figure 7C) and albumin (*R*^2^ = 0.98, Figure 7D). A similar linear relationship exists between IgG-LC and the mean injury score (*R*^2^ = 0.986, data not shown). In contrast, both BALF total protein (*R*^2^ = 0.999, Figure 7A) and E-Cadherin (*R*^2^ = 0.999, Figure 7B) demonstrated highly non-linear and accelerating relationships with mean injury score.

**Table 4:** Injury Scores from Histological Evaluation (*n* = 5 per group).

**Condition**	**Alveolar wall thickening**	**Alveolar wall rupture**	**Blood cell infiltration**	**Interstitial edema**	**Vascular bed injury**	**Alveolar wall flooding**
Low-Vt/PEEP0	0	0	0	0	0	0
Low-Vt/PEEP3	0	2	2	0	0	0
Mid-Vt/PEEP3	2	6–7	7	6–7	2–3	0
Mid-Vt/PEEP0	7–8	7–8	8	8	7–8	6–7
High-Vt/PEEP0	9–10	10	10	9–10	10	10

*Alveolar wall thickening: structural changes of the alveolar extracellular matrix (ECM) due to increased volume and/or loose ECM and hyaline membrane formation covering the epithelial layer. Alveolar wall rupture: thinner or broken alveolar walls with tissue retraction. Blood cell infiltration: increase of inflammatory cell number in the alveolar wall space. Interstitial edema: protein containing free fluid and swelling of the alveolar wall space ECM. Vascular bed injury: degeneration of vessels (swollen, vacuole rich vascular walls with cell necrosis). Alveolar wall flooding: platelet invasion including infarcts like hemorrhagic changes of alveolar walls after vascular breakage*.

## Discussion

Our results show that there is a strong relationship between the derecruitability of the injured lung (Figure 2B) and the magnitude of the alveolar leak as reflected in the amount (Figure 2D) and nature of material accumulated in the airspaces. Furthermore, we found that the elevated levels of BALF proteins that accumulate in an injured lung over time (Figures 3, 4, 5A) are strongly correlated with the appearance of retro-orbitally injected FITC-labeled dextran in the BALF (Figure 3A) and with histologic measures of alveolar septal injury (Figure 7). These results draw a tight link between altered lung function and the underlying pathologic process of epithelial injury and blood-gas barrier disruption. Such disruption could have manifested either as the appearance of gaps between the epithelial cells due to disruption of cell-cell tight junctions or as vacancies left in the epithelium as a result of cellular necrosis. In the latter case the destruction of the alveolar epithelium contributes directly to the accumulation of airspace protein. The level of soluble E-cadherin in the BALF, which is an indicator of both necrosis and barrier junction disruption of the epithelium (Thammanomai et al., 2013), increased linearly with BALF protein (Figure 4B), further confirming that the epithelial barrier is damaged proportionally with increased Vt.

Our study also demonstrates that the derecruitability test (Figure 2B and Supplementary Figure 2) provides a useful and sensitive means of monitoring the progress of injury itself. This concurs with our previous demonstration of a strong correlation between respiratory system elastance and the number of patent alveoli determined using design-based stereology in rats with bleomycin-induced lung injury (Lutz et al., 2015). The substantial injury to the Mid-Vt/PEEP0 and High-Vt/PEEP0 groups is evidenced by the fact that their *H*_1_ values were elevated compared to the pre-ventilation measurements at each PEEP level (Table 2). The elevated first elastance measurement at PEEP = 6 demonstrates that the recruitment maneuvers administered immediately prior to the derecruitability test were unable to fully open the lungs, presumably due to the effects of increased surface tension and the space-filling consequences of alveolar leak. The rates of elastance increase β^0^ and β^3^ were also elevated in the Mid- and High-Vt/PEEP0 groups, indicating that injury led to an increased degree of long-timescale derecruitment. However, β^6^ remained unchanged from pre-ventilation, suggesting injury did not increase derecruitment on the timescale of minutes at PEEP = 6 cmH_2_O. *H*_1_ values during the derecruitability tests decreased as PEEP increased from 0 to 3 cmH_2_O in all groups (Figure 2B, Table 3) reflecting increased recruitment with PEEP (Smith et al., 2015). At a derecruitment test PEEP = 6 cmH_2_O, however, $H_{1}^{6}$ increased in the pre-ventilation and PEEP = 3 cmH_2_O groups because of the volume-dependent stiffening of lung tissue (Smith et al., 2015). This did not happen in the PEEP = 0 ventilation groups, likely because there was so much airspace closure in this group that PEEP-dependent recruitment of lung units overwhelmed the effects of strain stiffening of the tissue.

Comparisons of pre- and post-ventilation derecruitability tests are possible under the controlled conditions of the laboratory. However, when assessing the severity and progression of clinical lung injury only the post-injury assessments of lung function may be available. Fortunately, the degree of injury can be inferred by comparing *H*_1_ and β at different derecruitability test PEEPs (Table 3). In the healthy (pre-ventilation) animals, $H_{1}^{0}>H_{1}^{6}>H_{1}^{3}$ and β remains constant between derecruitability test PEEPs. In the more severely injured lung the rate of elastance increase is greater at low PEEP so that β^0^ > β^3^ > β^6^. This difference in β is more pronounced with increasing injury severity as shown in the Mid- vs. High-Vt/PEEP0 groups in Table 3.

*H* (Figure 2B) and Δ*P*_*Max*_ (Figure 2A) are convenient mechanical indices for quantifying the degree of lung injury. $H_{1}^{0}$ exhibits a strong linear relationship with injury as reflected in BALF protein (Figure 2D), indicating that the amount of proteinaceous material accumulated in the airspaces leads to a proportional decrement during low-Vt ventilation. *P*_*S*_, in contrast, increases initially with increasing BALF protein but then plateaus at high levels of injury (Figure 2C), indicating that most airspace can still be recruited by a large breath even when large amounts of material have leaked in. However, altered peak pressures might also reflect the interference of proteinaceous debris with dynamic surfactant function, because interfacial forces play an important role in the alveolar pressure-volume relationship (Bachofen et al., 1970). The exception to this relationship is the Low-Vt/PEEP0 group in which elevated values of *P*_*S*_ did not correspond to substantial increases in BALF protein (Figure 2C). This is because the elevated Δ*P*_*Max*_ values in this group were due merely to derecruitment of airspaces that were readily re-recruited with a deep lung inflation as demonstrated in the derecruitment tests (Figure 2B). Conversely, the progressive increases seen in the Mid-Vt/PEEP0 and High-Vt/PEEP0 groups were due to actual damage to the lung tissues as evidenced by the histological scoring (Table 4). These findings show that *H*_1_ and *P*_*S*_ convey complementary information and are thus more useful together than individually.

In order to gain further insight into the source of the BALF protein content we examined its components with focus on species with blood or epithelial origins. As shown by western blot analysis (Figure 4), the blood-borne molecules albumin, IgG-HC, and IgG-LC were increased proportionally by injury. This behavior was also reflected in gel staining (Figure 3) with greater increases in medium-sized (65–70 kDa) proteins (most likely corresponding to albumin) than large (150–250 kDa) proteins during injurious ventilation. This suggests that the perforations in the epithelial barrier caused by injurious ventilation acted like a sieve at moderate levels of injury by impeding the passage of larger molecules, which is consistent with previous reports of an increase in effective pore size as lung volume is increased (Egan et al., 1976; Yoshikawa et al., 2004). When injury is severe, the sieve effect might disappear; ARDS patients with BALF protein concentrations equivalent to our High-Vt/PEEP0 group were found to have plasma macromolecules in the airspaces in amounts proportional to their plasma concentrations (Holter et al., 1986). Histological scoring (Table 4) provides further support for this hypothesis. Although, the mean injury score was linearly correlated with BALF concentrations of IgG-HC (Figure 7C), albumin (Figure 7D), and IgG-LC (*R*^2^ = 0.986, data not shown), BALF total protein content (Figure 7A) and E-Cadherin (Figure 7B) exponentially increased with mean injury score in a way that is indicative of an epithelial injury threshold for the ingress of larger plasma proteins. However, the linear correlation between E-Cadherin and total protein might also be indicative of the accumulation of airspace proteins from injured epithelial cells, and the large BALF proteins might have originated in epithelial cells destroyed by the mechanical forces of ventilation.

As we have found previously, producing substantial lung injury in initially healthy mice required that they be ventilated with both large Vt and zero PEEP, as in the Mid-Vt/PEEP0 and High-Vt/PEEP0 groups (Figures 2–4). Equivalent levels of distention with PEEP = 3 cmH_2_O, as in the Mid-Vt/PEEP3 group, did not produce measurable changes in either peak pressures (Figure 2A and Supplementary Table 1) or derecruitability (Figure 2B and Supplementary Tables 2–4) and resulted in only a very small elevation in the level of E-Cadherin in the BALF (Figure 5A). Once VILI was initiated at PEEP = 0, however, the rate and degree of injury could be titrated by choice of Vt (compare the Mid-Vt/PEEP0 and High-Vt/PEEP0 groups in Figures 2–4). In the mouse model employed in the present study, PEEP = 3 cmH_2_O was sufficient to avoid the beginning of atelectrauma and thus prevented progression to VILI (Smith et al., 2013), at least over the 100 min duration of the experiment. By contrast, PEEP = 0 did not prevent the onset of atelectrauma and the associated wounding of alveolar epithelial cell plasma membranes (Hussein et al., 2013).

It should also be noted that the Vt we used in our mice (10–44 ml/kg delivered to the animal after taking cylinder gas compression into account) are substantially greater than those ever used clinically, as is frequently the case in mice (Wilson et al., 2012). These tidal volumes all exceeded the 6 ml/kg target based on the ARDSnet trial (Acute Respiratory Distress Syndrome Network, 2000). However, these high tidal volumes in patent lungs are representative of the high regional strains occurring during clinical ventilation of highly derecruited “baby” lungs (Gattinoni and Pesenti, 2005). Furthermore, the presence of heterogeneous collapse may amplify strain on the alveolar scale by creating stress concentrations around atelectatic alveoli (Mead et al., 1970; Makiyama et al., 2014). Our choice of Vt is also influenced by previous studies where we observed that initially healthy mice do not develop VILI over an experimentally tractable period unless Vt well above the clinical guidelines are used (Seah et al., 2011; Smith et al., 2013). The difference in delivered tidal volume between groups required that we either adjust respiratory rate in the Mid- and High-Vt to provide equal minute ventilation or use a consistent respiratory rate in order to provide an equal frequency of mechanical injury application. Because of our focus on the mechanical aspects of injury we elected to maintain a consistent respiratory rate despite the fact this altered the minute ventilation between groups (Low-Vt ≈ 2.0 L/kg/min, Mid-Vt/PEEP3 = 1.6 L/kg/min, Mid-Vt/PEEP0 = 1.7 L/kg/min, High-Vt/PEEP0 = 2.2 L/kg/min). In light of observations that high CO_2_ levels impair alveolar epithelial fluid clearance (Briva et al., 2007), the reduced minute ventilation in the Mid-Vt groups might have enhanced injury.

**Figure 8:**
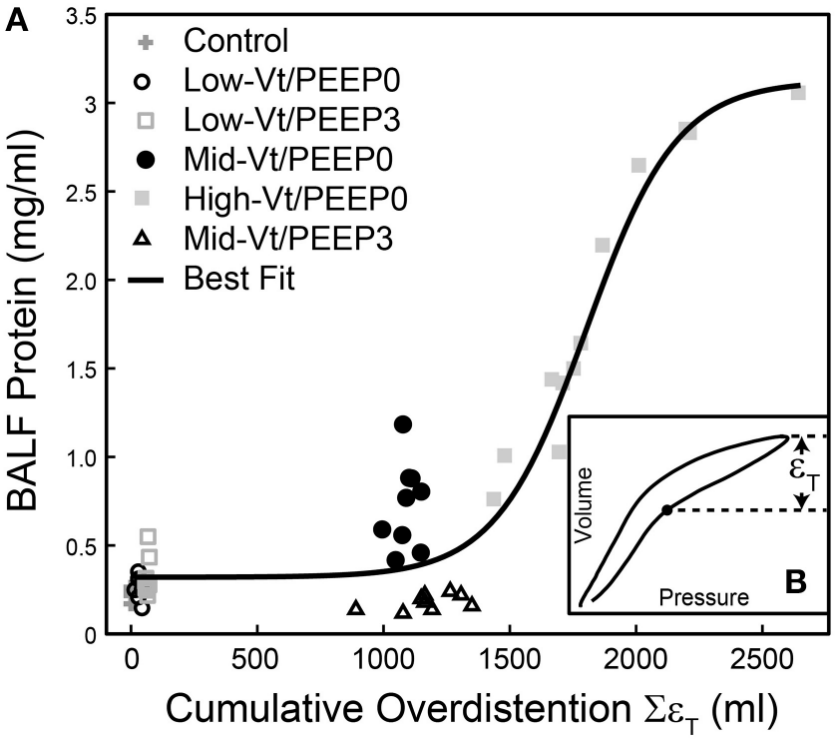
Bronchoalveolar lavage fluid (BALF) total protein plotted against the total cumulative lung overdistension (Sε_T_) for the six experimental groups **(A)**. The sigmoidal best fit (black line) *y* = 0.32 + 2.80/(1 + e^−(x-1802.2)/180.8^), *R*^2^ = 0.92, *p* < 10^−6^. Sε_T_ is quantified as the total volume delivered over the upper corner point [determined by fitting the Venegas equation (Venegas et al., 1998) as described in Section Pressure-Volume Loop Analysis] shown with a filled circle in inset **(B)** and in Figure 1.

We investigated the link between VILI and tissue overdistension further by comparing an estimate of ventilator-induced tissue strain vs. BALF protein for the various study groups. Figure 8 shows this relationship, where cumulative injurious tissue strain has been estimated as the total volume delivered above the upper corner point on the pressure-volume curve. The relationship in Figure 8 demonstrates a threshold volume for overdistension injury that bears striking similarity to the strain threshold for edema reported by Protti et al. (2011) in pigs. This pig model was protected from injury by the addition of PEEP (Protti et al., 2013) despite the presence of extremely high levels of lung distention, further supporting our findings that the normal lung is highly resistant to volutrauma alone. In other words, atelectrauma and volutrauma together are required to produce VILI (Seah et al., 2011). The important role of atelectrauma in epithelial injury has also been demonstrated in isolated rat lungs following the instillation of a small amount of saline. Significant wounding of the epithelial cell plasma membrane occurred during ventilation with PEEP = 0, but this was abrogated by the addition of PEEP (Hussein et al., 2013). Similarly, total liquid ventilation has been shown to reduce epithelial damage substantially by removing the effects of surface tension altogether, along with the damaging effects of cyclic recruitment (Hussein et al., 2013).

However, the importance of volutrauma in activating inflammation cannot be overlooked. For example, pigs subjected to a saline lavage and Low-Vt/High-PEEP ventilation (volutrauma) demonstrated a marked increase in inflammation that was not present in animals receiving Low-Vt/Low-PEEP ventilation (atelectrauma) (Güldner et al., 2016). Further support for the role of volutrauma in activating the inflammatory response is provided in the increased monocyte activation and proinflammatory cytokine levels observed in isolated perfused mouse lungs ventilated at high Vt and PEEP = 3 cmH_2_O when comparted to low-Vt ventilation at PEEP = 0 and 5 cmH_2_O (Wakabayashi et al., 2014).

Our findings might also be taken to suggest that the applied ventilation causes endothelial injury, because blood-derived fluid and proteins must first leak out of the vasculature before being able to traverse the epithelium. On the other hand, pulmonary endothelial leak is regulated naturally as a means of controlling egress of immune cells and mediators at sites of inflammation and infection, so some degree of baseline leak is perhaps normal. Build-up of hydrostatic pressure within the interstitium of the lung would then be the main impediment to continued exit of fluid and protein under normal conditions. Release of interstitial pressure due to epithelial leak would then allow continued efflux of material from the blood. Thus, direct damage to the endothelium by atelectrauma might not be a necessity for VILI to develop, at least during its early stages.

One possible scenario that might explain the synergy between volutrauma and atelectrauma is that injury begins when the high tissue strains and/or strain rates associated with a sufficiently large Vt produce a relatively minor and size-selective epithelial leak (Egan, 1980; Yoshikawa et al., 2004). The material that enters the airspaces via this leak (and due to the necrosis of epithelial cells) then begins to degrade surfactant function, which, along with tidal volume-induced surfactant inactivation (Mascheroni et al., 1988; Veldhuizen et al., 2002), causes an elevation in the pressures at which the affected lung units recruit and derecruit. The repetitive re-recruitment of these units that starts to occur with each breath is itself extremely damaging to the epithelium (Bilek et al., 2003; Kay et al., 2004; Glindmeyer et al., 2012; Hussein et al., 2013), which worsens the leak and increases the damaging stresses and strains on the tissue in a vicious cycle. Other factors might also be operative, however. For example, high Vt imposed on a derecruited lung is likely to exacerbate septal overdistension caused by alveolar interdependence (Mead et al., 1970; Makiyama et al., 2014). That is, the collapse of one alveolus increases the distension in adjacent patent alveoli, something that has recently been shown to increase leak in the adjacent alveoli (Wu et al., 2014). If, in addition, there is a large difference between the pressures at which lung units open and close, a large Vt is more likely to visit both pressures and thus cause recruitment and derecruitment throughout the breath, whereas a small Vt will not. Such an effect might, for example, explain why reduced Vt has been found to be protective in ARDS (Acute Respiratory Distress Syndrome Network, 2000).

Finally, while the present study arguably advances our understanding of VILI by linking potentially clinically observable manifestations to the underlying processes responsible for these manifestations, the study findings must be viewed in light of its limitations. Most important is the question of how well data obtained in mice translate to the human patient. Although, there is no particular reason to think that stresses and strains that are injurious to a mouse lung would not also be injurious to a human, we cannot be sure this is the case. Also, the ventilatory maneuvers we performed on our mice to assess their lung function and derecruitability might well not be ethical for a patient with ARDS; such assessments would likely have to be done by employing more subtle maneuvers in patients, which could affect the quality of the data obtained. Another limitation of our study is that we generated VILI by applying injurious ventilation to initially normal lungs. This proved useful in an animal model because it allowed VILI to be generated in a controlled and titratable manner, but it does not represent a clinically relevant scenario. The various ventilation regimens also resulted in up to a 37.5% variation in minute ventilation between the various animal groups studied. This would have likely resulted in somewhat different alveolar PCO_2_ values between the groups that, while probably not large compared to those seen in ARDS patients subjected to permissive hypercapnia compared to controls (Tuxen, 1994), may still have had physiological effects on inflammatory processes and smooth muscle activity. It thus remains to be seen how well our findings translate to VILI in lungs that are already injured in ways typical of the ARDS patient, such as via sepsis or aspiration.

In summary, we have shown that alterations in lung mechanics, particularly as revealed by the derecruitability test, are sensitive reflections of the degree of damage to the epithelial barrier through which blood-derived materials make their way into the airspaces where they give rise to the most clinically obvious phenotype of acute lung injury. VILI can thus be seen as a dynamic process in which initial injury begets leak, and thus more injury, in a downward spiral that manifests as an accelerating decline in lung mechanical function. The most important step in preventing this downward spiral would appear to be avoidance of cyclic recruitment and derecruitment before significant damage has been inflicted on the epithelial barrier. Our findings also indicate that the ongoing measurements of lung mechanics, particularly those related to the dynamic derecruitability of the lung, provide the most sensitive and effective means for rapidly tracking the evolution of VILI, suggesting that it would be useful to explore ways of making such measurements in ARDS patients.

## Author contributions

BJS, JB, GR, EB, BS, and KH contributed to the design of the study. BJS, EB, GR, and JB prepared and drafted the manuscript. BJS, JB, GR, EB, BS, and KH interpreted the experimental data and performed critical revision of the manuscript for intellectual content. BJS, CC, GR, KH, and EB carried out the experimental and histological studies.

### Conflict of interest statement

BJS and JB have a patent pending entitled “Variable ventilation as a diagnostic tool for assessing lung mechanical function.” JB and BJS PCT Application WO2015127377 A1, Filed on February 23, 2014 (C538). JB has a patent pending entitled “Device and method for lung measurement” JB U.S. Patent application US 20160007882 A1, Filed on January 29. 2013. JB is a scientific advisor for and minor shareholder of Oscillavent, LLC. The other authors declare that the research was conducted in the absence of any commercial or financial relationships that could be construed as a potential conflict of interest.
