# Supplementary material for: Linking Ventilator Injury-Induced Leak across the Blood-Gas Barrier to Derangements in Murine Lung Function
Source: Front Physiol. 2017 Jul 7;8:466. doi: 10.3389/fphys.2017.00466 (PMC5500660; doi:10.3389/fphys.2017.00466)
Supplement: Supplementary file 1 [file DataSheet1.pdf]

frontiers

Supplementary Material

Linking Ventilator Injury-Induced Leak Across the Blood-Gas Barrier to Derangements in Murine Lung Function

Bradford J. Smith*, Elizabeth Bartolak-Suki, Bela Suki, Gregory S. Roy, Katharine L. Hamlington, Chantel Charlebois, and Jason H. T. Bates

*** Correspondence:** Bradford.Smith@ucdenver.edu

1 Supplementary Tables

Supplementary Table 1: Mean differences between ventilation groups [95% CI] † in the slope (P_S) of the linear fit to the pressure-volume loop peak pressure (P_{Max}) recorded during ventilation.

	Mid-Vt/PEEP0	Low-Vt/PEEP0	Mid-Vt/PEEP3	Low-Vt/PEEP3
High-Vt/ PEEP0	0.052 [0.013, 0.090]*	0.097 [0.063, 0.131]*	0.127 [0.102, 0.153]*	0.129 [0.103, 0.154]*
Mid-Vt/ PEEP0		0.045 [0.006, 0.084]*	0.076 [0.041, 0.110]*	0.077 [0.042, 0.111]*
Low-Vt/ PEEP0			0.030 [0.003, 0.058]*	0.032 [0.004, 0.059]*
Mid-Vt/ PEEP3				0.001 [−0.007, 0.010]

†Differences are between row − column. *Shading indicates statistical significance at p<0.05.

Supplementary Table 2. Comparison of the effect of ventilation treatment on elastance measured during PEEP = 0 cmH_2O derecruitability tests showing the mean differences [95% CI] † in initial elastance (H_1) and in the elastance increase rate (β) between groups.

PEEP 0		Mid-Vt/PEEP0	Low-Vt/PEEP0	Mid-Vt/PEEP3	Low-Vt/PEEP3
High-Vt/ PEEP0	H_1^0	17.24 [−1.38, 35.85]	20.80 [2.28, 39.32]*	21.29 [2.83, 39.76]*	21.43 [2.99, 39.87]*
	β^0	0.018 [−0.017, 0.052]	0.042 [0.012, 0.071]*	0.042 [0.013, 0.071]*	0.048 [0.019, 0.076]*
Mid-Vt/ PEEP0	H_1^0		3.56 [−1.50, 8.62]	4.06 [−0.64, 8.75	4.20 [−0.40, 8.80]
	β^0		0.024 [−0.003, 0.051]	0.025 [−0.001, 0.050]	0.030 [0.005, 0.056]*
Low-Vt/ PEEP0	H_1^0			0.50 [−2.96, 3.95]	0.64 [−2.62, 3.89]
	β^0			0.001 [−0.014, 0.016]	0.006 [−0.008, 0.021]
Mid-Vt/ PEEP3	H_1^0				0.14 [−1.84, 2.12]
	β^0				0.006 [−0.003, 0.014]

†Differences are between row − column. *Shading indicates statistical significance at p<0.05.

Supplementary Table 3. Comparison of the effect of ventilation treatment on elastance measured during PEEP = 3 cmH_2O derecruitability tests showing the mean differences [95% CI] † in initial elastance (H_1) and in the elastance increase rate (β) between groups.

PEEP 3		Mid-Vt/PEEP0	Low-Vt/PEEP0	Mid-Vt/PEEP3	Low-Vt/PEEP3
High-Vt/	H_1^3	10.24 [0.44, 20.03]*	14.13 [4.21, 24.05]*	13.56 [3.73, 23.39]*	14.45 [4.70, 24.20]*
PEEP0	β^3	−0.001 [−0.024, 0.022]	0.011 [−0.006, 0.027]	0.011 [−0.002, 0.024]	0.014 [0.002, 0.027]*
Mid-Vt/	H_1^3		3.89 [0.40, 7.39]*	3.32 [0.48, 6.16]*	4.21 [2.04, 6.38]*
PEEP0	β^3		0.012 [−0.012, 0.035]	0.012 [−0.010, 0.035]	0.015 [−0.007, 0.037]
Low-Vt/	H_1^3			−0.57 [−4.23, 3.09]	0.32 [−2.99, 3.63]
PEEP0	β^3			0.001 [−0.013, 0.015]	0.004 [−0.010, 0.017]
Mid-Vt/	H_1^3				0.89 [−1.64, 3.43]
PEEP3	β^3				0.003 [−0.005, 0.011]

†Differences are between row − column. *Shading indicates statistical significance at p<0.05.

Supplementary Table 4. Comparison of the effect of ventilation treatment on elastance measured during PEEP = 6 cmH_2O derecruitability tests showing the mean differences [95% CI] † in initial elastance (H_1) and in the elastance increase rate (β) between groups.

PEEP 6		Mid-Vt/PEEP0	Low-Vt/PEEP0	Mid-Vt/PEEP3	Low-Vt/PEEP3
High-Vt/	H_1^6	7.29 [−1.83, 16.42]	12.63 [3.95, 21.31]*	10.62 [1.98, 19.27]*	13.06 [4.54, 21.57]*
PEEP0	β^6	0.005 [−0.008, 0.017]	−0.002 [−0.013, 0.010]	0.008 [−0.011, 0.027]	0.005 [−0.002, 0.013]
Mid-Vt/	H_1^6		5.34 [−0.26, 10.93]	3.33 [−2.19, 8.85]	5.76 [0.50, 11.03]*
PEEP0	β^6		−0.007 [−0.021, 0.008]	0.003 [−0.017, 0.023]	0.000 [−0.012, 0.012]
Low-Vt/	H_1^6			−2.00 [−6.25, 2.23]	0.43 [−3.35, 4.20]
PEEP0	β^6			0.009 [−0.010, 0.029]	0.007 [−0.004, 0.018]
Mid-Vt/	H_1^6				2.43 [−1.17, 6.03]
PEEP3	β^6				−0.003 [−0.021, 0.016]

†Differences are between row − column. *Shading indicates statistical significance at p<0.05.

Supplementary Table 5. Post-ventilation derecruitability test initial elastance (H_1) showing the mean, standard deviation (SD), 95% confidence intervals (CI), the minimum (Min), and maximum (Max).

		Mean	SD	95% CI	Min	Max
PEEP = 0 cmH_2O	Low-Vt/PEEP0	23.35	2.82	[21.18, 25.52]	19.55	26.80
	Mid-Vt/PEEP0	26.91	3.99	[23.84, 29.98]	22.26	33.11
	High-Vt/PEEP0	44.15	19.75	[31.60, 56.70]	23.07	88.98
	Mid-Vt/PEEP3	22.86	1.70	[21.55, 24.16]	19.46	24.83
	Low-Vt/PEEP3	22.72	0.59	[22.30, 23.13]	21.95	23.48
PEEP = 3 cmH_2O	Low-Vt/PEEP0	15.75	2.84	[13.56, 17.93]	11.83	19.66
	Mid-Vt/PEEP0	19.64	1.64	[18.27, 21.01]	17.31	21.97
	High-Vt/PEEP0	29.88	10.43	[23.25, 36.50]	17.28	44.49
	Mid-Vt/PEEP3	16.32	2.13	[14.68, 17.96]	13.93	20.26
	Low-Vt/PEEP3	15.43	1.11	[14.63, 16.22]	14.28	17.74
PEEP = 6 cmH_2O	Low-Vt/PEEP0	18.67	3.03	[16.35, 21.00]	15.39	24.43
	Mid-Vt/PEEP0	24.01	4.46	[20.58, 27.44]	20.59	32.89
	High-Vt/PEEP0	31.31	9.01	[25.58, 37.03]	17.27	44.11
	Mid-Vt/PEEP3	20.68	2.84	[18.50, 22.86]	15.34	25.39
	Low-Vt/PEEP3	18.25	2.14	[16.71, 19.78]	15.45	21.98

Supplementary Table 6. Post-ventilation derecruitability test elastance increase rate (β) showing the mean, standard deviation (SD), 95% confidence intervals (CI), the minimum (Min), and maximum (Max).

	Post-Ventilation β PEEP 0	Mean	SD	95% CI	Min	Max
PEEP = 0 cmH_2O	Low-Vt/PEEP0	0.0354	0.0124	[0.0258, 0.0449]	0.0189	0.0618
	Mid-Vt/PEEP0	0.0593	0.0220	[0.0424, 0.0762]	0.0377	0.1001
	High-Vt/PEEP0	0.0768	0.0305	[0.0575, 0.0962]	-0.0066	0.1105
	Mid-Vt/PEEP3	0.0347	0.0074	[0.0290, 0.0403]	0.0213	0.0439
	Low-Vt/PEEP3	0.0290	0.0038	[0.0263, 0.0317]	0.0241	0.0346
PEEP = 3 cmH_2O	Low-Vt/PEEP0	0.0348	0.0117	[0.0258, 0.0438]	0.0230	0.0622
	Mid-Vt/PEEP0	0.0464	0.0175	[0.0318, 0.0610]	0.0302	0.0798
	High-Vt/PEEP0	0.0454	0.0129	[0.0372, 0.0536]	0.0331	0.0703
	Mid-Vt/PEEP3	0.0340	0.0063	[0.0292, 0.0388]	0.0231	0.0427
	Low-Vt/PEEP3	0.0313	0.0047	[0.0279, 0.0347]	0.0254	0.0399
PEEP = 6 cmH_2O	Low-Vt/PEEP0	0.0325	0.0094	[0.0252, 0.0397]	0.0212	0.0480
	Mid-Vt/PEEP0	0.0259	0.0103	[0.0180, 0.0339]	0.0049	0.0401
	High-Vt/PEEP0	0.0308	0.0068	[0.0265, 0.0352]	0.0214	0.0451
	Mid-Vt/PEEP3	0.0232	0.0162	[0.0107, 0.0357]	-0.0085	0.0413
	Low-Vt/PEEP3	0.0257	0.0046	[0.0224, 0.0290]	0.0188	0.0318

2 Supplementary Figures

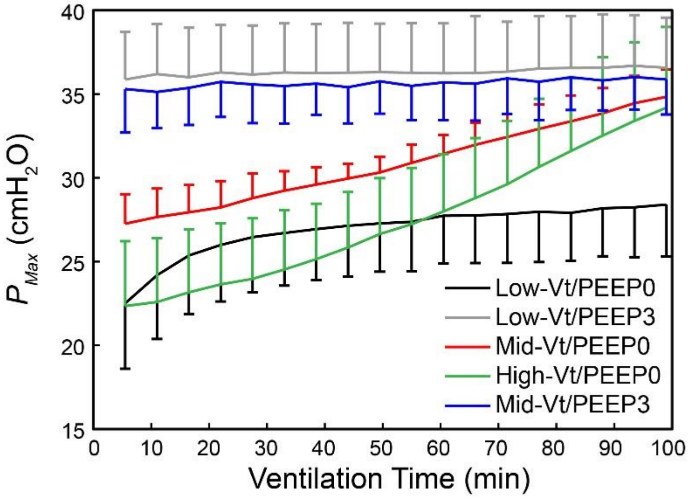

Supplementary Figure 1: Dynamic pressure-volume loop peak pressure (P_{Max}) during ventilation showing the mean and standard deviation for each ventilation treatment group.

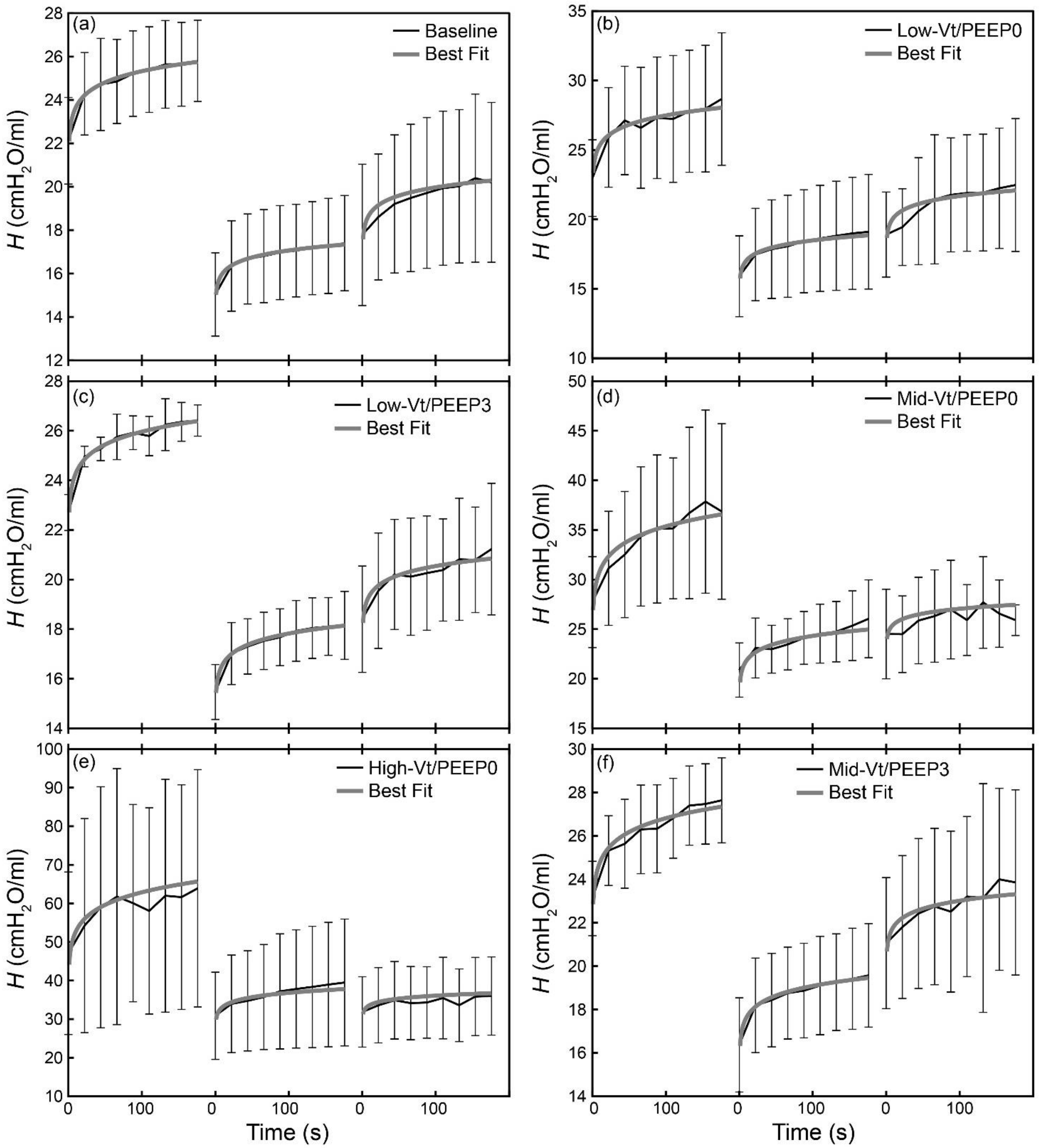

Supplementary Figure 2: Pulmonary system elastance (*H*) measured during derecruitability tests. Baseline measurements (a) show the average of all groups prior to ventilation. (b)-(f) show derecruitment dynamics following ventilation. The best-fit line (grey) has the form y = $H_1\ t^{\beta}$ where *t* is the time in the derecruitability test. Values for H_1 and β are given in Supplementary Tables 5 and 6.

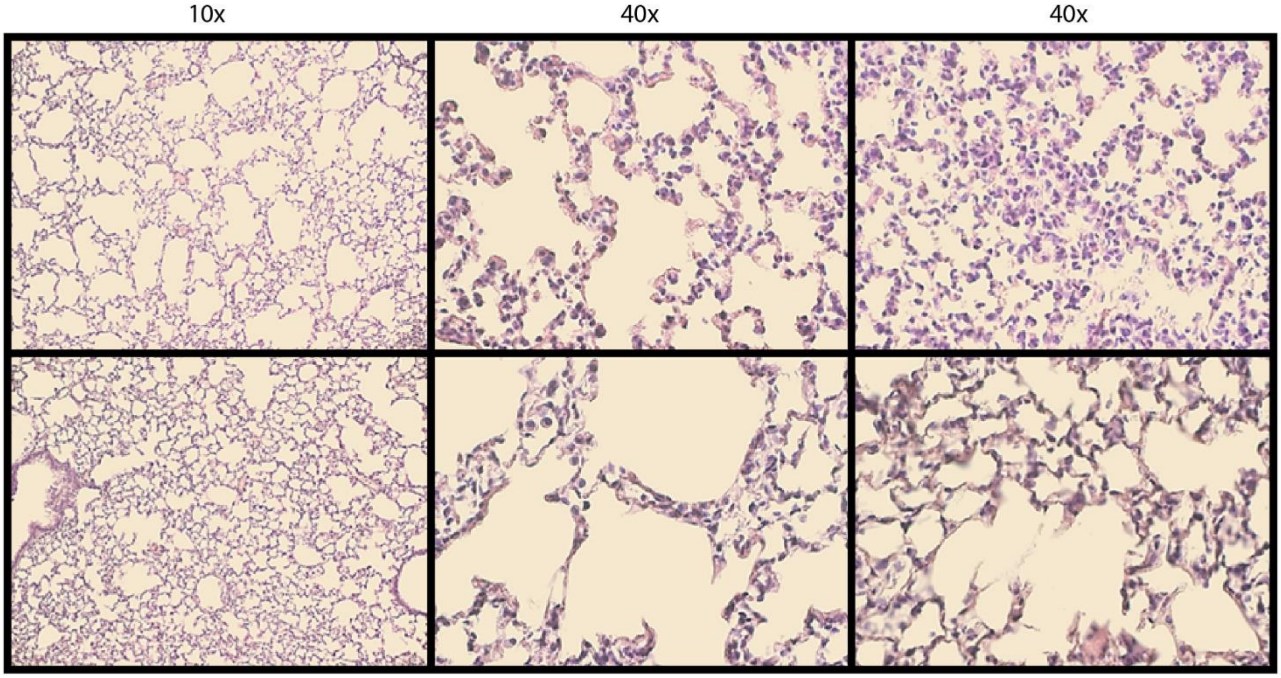

Supplementary Figure 3: Representative micrographs of hematoxylin and eosin (H&E) stained mouse lung sections at Mid-Vt/PEEP0 ventilation. 10x magnification shows heterogeneous airspace enlargement with varying degrees of collapse. The top two panels at 40x show tissue degeneration in these areas including epithelial and endothelial cell necrosis. Hyaline membranes (pink sheets) cover the epithelial alveolar layer. The bottom two panels demonstrate weakened walls with some interstitial edema but less degeneration than in High-Vt/PEEP0.

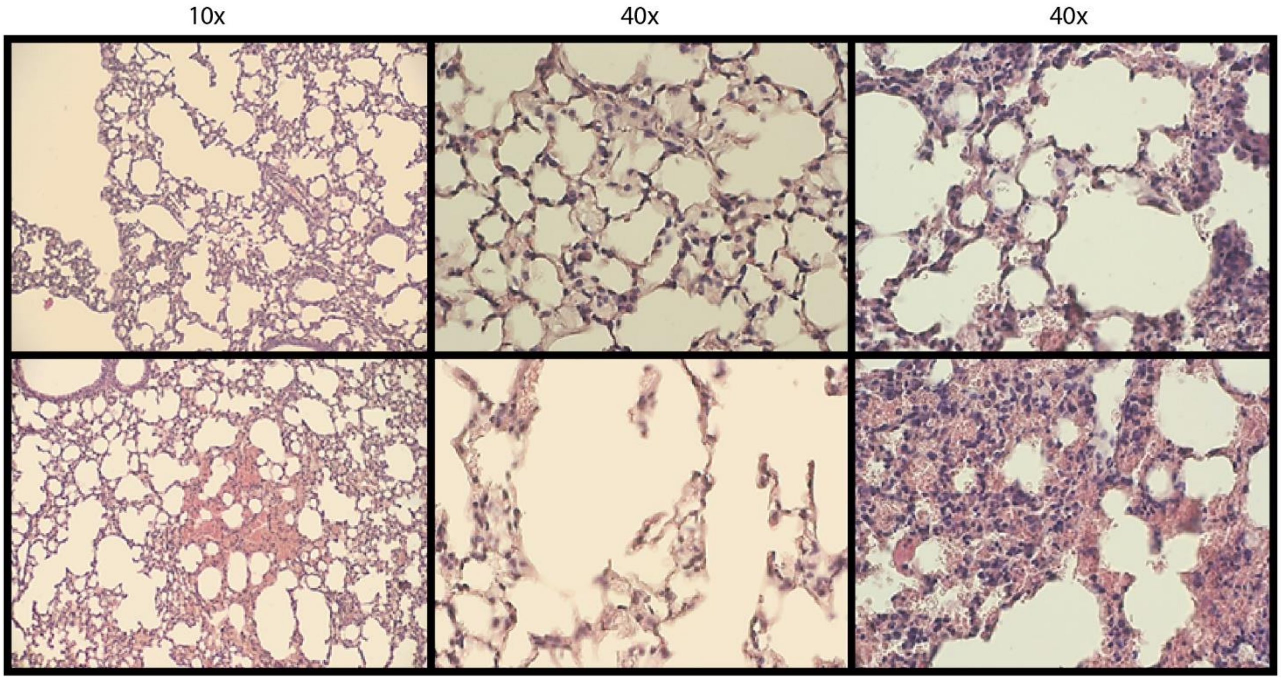

Supplementary Figure 4: Representative H&E sections at High-Vt/PEEP0 ventilation. At 10X magnification more advanced degeneration of the alveolar walls is evident in this group than in the Mid-Vt/PEEP0 group (left) with areas of hemorrhage (right). The top two panels at 40X magnification show less tissue degeneration than the bottom two panels. These images show more interstitial edema and thicker hyaline membranes (pink sheets covering the epithelial alveolar layer) than was found in the Mid-Vt/PEEP0 group. On the bottom two panels more tissue damage is present with broken vessel walls due to cell necrosis; hence, the alveoli are flooded with blood cells causing pulmonary infarction (hemorrhagic).
